# Healthcare needs, expectations, utilization, and experienced treatment effects in patients with hereditary spastic paraplegia: a web-based survey in the Netherlands

**DOI:** 10.1186/s13023-021-01915-0

**Published:** 2021-06-24

**Authors:** Hans C. J. W. Kerstens, Bas J. H. Van Lith, Maarten J. Nijkrake, Bert J. M. De Swart, Laura A. C. Van den Bemd, Rob J. E. M. Smeets, Fheodoroff Klemens, Bart P. C. Van de Warrenburg, Philip J. Van der Wees, Alexander C. H. Geurts

**Affiliations:** 1 IQ Healthcare, Radboud Institute for Health Sciences, Radboud University Medical Center, Nijmegen, The Netherlands; 2 Department of Rehabilitation, Donders Institute for Brain, Cognition and Behaviour, Radboud University Medical Center, Nijmegen, The Netherlands; 3 HAN University of Applied Sciences, Nijmegen, The Netherlands; 4 Department of Rehabilitation, Sint Maartenskliniek, Nijmegen, The Netherlands; 5 Department of Rehabilitation Medicine, Maastricht University, Research School CAPHRI, Maastricht, The Netherlands; 6 CIR Revalidatie, Eindhoven, The Netherlands; 7 Department of Neurorehabilitation, Gailtal-Klinik, Hermagor, Austria; 8 Department of Neurology, Donders Institute for Brain, Cognition and Behaviour, Radboud University Medical Center, Nijmegen, The Netherlands

**Keywords:** Hereditary spastic paraplegia, Spasticity, Survey, Patient-reported outcomes, Needs assessment, Healthcare utilization, Experienced treatment effects

## Abstract

**Background:**

We aimed to identify healthcare needs, expectations, utilization, and the experienced treatment effects in a population of Dutch patients with hereditary spastic paraplegia (HSP).

**Methods:**

We distributed an online questionnaire among 194 adult persons with HSP in the Netherlands, of which 166 returned a fully completed version. After applying predefined exclusion criteria, 109 questionnaires from persons with pure HSP were analysed.

**Results:**

Healthcare needs and expectations were primarily focused on the relief of muscle stiffness and reduction of balance and gait impairments (65–80%), but many participants also expressed needs regarding relief of non-motor symptoms (e.g. pain, fatigue), emotional problems, impaired sleep and self-care capacity, and participation problems (> 60%). Remarkably, despite these frequent needs, relatively few participants (< 33%) expected to be able to improve in these additional domains. Rehabilitation physicians and physiotherapists were more frequently consulted than neurologists and occupational therapists, respectively. Physiotherapy was the most often proposed non-pharmacological intervention (85%), followed by orthopedic footwear (55%) and splints (28%). Approximately one third of the participants was never offered any pharmacological (spasmolytic) treatment. Spasmolytic oral drugs, injections, and intrathecal baclofen were given to 41%, 26%, and 5% of the participants, respectively. Independent of the type of pharmacological intervention, 35–46% of these participants experienced decreased spastiticy and improved general fitness. Other experienced effects differed per type of intervention.

**Conclusions:**

Based on this web-based survey in the Netherlands, there seems to be ample room for improvement to meet and attune the healthcare needs and expectations of people with HSP concerning both their motor and non-motor symptoms and functional limitations. In addition, the provision of adequate information about non-pharmacological and pharmacological interventions seems to be insufficient for many patients to allow shared decision making. These conclusions warrant a more pro-active attitude of healthcare providers as well as an interdisciplinary approach for a substantial proportion of the HSP population, also involving professionals with a primary occupational and/or psychosocial orientation.

**Supplementary Information:**

The online version contains supplementary material available at 10.1186/s13023-021-01915-0.

## Background

Hereditary spastic paraplegia (HSP) is a group of inherited neurological disorders characterized by progressive bilateral lower limb spasticity and muscle weakness [1]. Many complex forms of HSP exist, but in patients with ‘pure HSP’ the main neurological feature is a slowly progressive spastic paraparesis [2–4]. In a previous publication [5], we reported the first results of a web-based survey in the Netherlands, focusing on the spasticity-related complaints and activity limitations as experienced by patients with HSP. These data showed that patients with pure HSP experienced the greatest burden from muscle stiffness, physical and mental fatigue, leg and back pain, and limitations with regard to standing and walking activities. Furthermore, they reported a high frequency of walking aid use, fall incidents, and fear of falling. These findings are in line with the results of a previously published, qualitative study amongst persons with pure HSP [6]. Additionally, the participants in this qualitative study reported that they often missed the support from healthcare professionals in dealing with their spasticity-related complaints and activity limitations. More specifically, they expressed a need for personalized guidance and advice on how to adjust to the consequences of HSP in everyday life [6]. In their Cochrane review on multidisciplinary treatment following focal spasmolysis in people post stroke, Demetrios et al. stated that “using appropriate patient-centered outcomes of rehabilitations interventions with standardized measures may provide a more holistic picture” [7]. This was the reason to also include specific questions about healthcare needs, expectations, and utilization in the design of our web-based survey. In the present study, we focus on these specific questions.

Although similar healthcare aspects have already been investigated in an international survey of a mixed patient population living with more common causes of spasticity (e.g. stroke, multiple sclerosis, traumatic brain injury) [8], these issues have not yet been investigated in patients with inherited and progressive forms of spastic paraparesis. Furthermore, the experienced treatment effects reported in the abovementioned international survey were solely focused on botulinum toxin injections, not on pharmacological treatment in general.

Hence, in the present study, we report the data from our web-based survey addressing the healthcare needs, expectations, utilization, and experienced treatment effects amongst patients with pure HSP in the Netherlands [5]. The specific research questions were: (1) What needs do patients report regarding the symptoms and consequences of spastic paraparesis? (2) What treatment effects do patients expect at forehand? (3) Which healthcare professionals do they consult and how often? (4) Which interventions are proposed by their healthcare providers? and (5) What pharmacological treatment effects do they experience?

## Methods

### Study design and setting

An online survey was conducted between January 2017 and June 2017 in the Netherlands amongst community dwelling persons with HSP. The content of the survey aimed to identify the experienced consequences of living with inherited and progressive spastic paraparesis, as well as the experienced needs and expectations regarding clinical management. As the data on the experienced consequences have been reported in our previous publication [5], the current study focused on the healthcare needs, expectations, utilization and experienced effects regarding clinical management. The study was approved by the regional medical ethics committee “Commissie Mensgebonden Onderzoek Arnhem-Nijmegen” (number: 2016-2922), and conducted according to the declaration of Helsinki.

### Participants

Participants were recruited in three different ways. First, on our request, the national patient organization for neuromuscular disorders in the Netherlands (‘Spierziekten Nederland’; www. spierziekten.nl) invited their members of the HSP working group by sending an email with information about the web-based survey. Second, all patients with pure HSP known at the expert center for rare and genetic movement disorders of the Radboud University Medical Center in Nijmegen were sent a letter with information about the survey. Third, all people to whom we reached out by either of the above-mentioned ways were asked to share their invitation with relatives suffering from HSP. All invited persons and relatives could then apply to participate by sending an email to one of the researchers (BvL). After receiving an email in which the person stated to be willing to participate, BvL sent a unique link to the web-based questionnaire to each potential participant. People were eligible if they were 18 years or older and had genetically confirmed HSP or, according to the recruiting researcher (BvL) and the senior author (AG), were very likely to have HSP based on their clinical symptoms and family history.

### Data collection

The structure and content of the survey were designed by a team of expert physicians, physical therapists, researchers, and persons with HSP, as described in our previous publication [5]. Part of the questionnaire (category D) was based on a previous international survey amongst patients living with spasticity [8]. This was extended with questions based on the findings from a qualitative study amongst patients living with pure HSP, who were interviewed about the daily life consequences of spastic paraparesis and related healthcare needs [6]. In addition, representatives of the national patient organization were consulted. The survey was sent to the participants using Castor electronical data capture (Castor EDC v2020.1.15).

The content of the survey was categorized in four categories: A. participant characteristics, B. complaints and activity limitations, C. loss of motor capacities, and D. healthcare needs and interventions. For the current study, only the categories A and D were used. The posed questions and the accompanying answering categories are listed in the additional file 1. To some extent, the amount of questions was variable for each participant, depending on their answer to a preceding question. Answering options were based on multiple choice, but some questions included a free entry format as one of the options. Completion of all questions took about 20 min, but there was no set time limit. Participants were allowed to pause during the questionnaire and continue at a later moment in time.

To ensure the inclusion of a homogeneous sample of persons with pure HSP, specific questions were included to identify patients with a complicated form of HSP and/or with neurological comorbidity.

### Data analysis

We excluded persons who confirmed that they had a complicated form of HSP (i.e. a genetic defect invariably associated with a complicated form of HSP and/or upper limb paresis, speech problems, cognitive disorders); or stated to have any neurological comorbidity that might interfere with spasticity, motor control, or physical fitness and activity. Furthermore, we excluded persons who reported that they suffered from spasticity for less than one year because of their limited experience with clinical management.

Data was exported from Castor into Excel files, which were imported into a statistical software program. Descriptive statistics were run in SPSS (IBM Corp. Released 2017, IBM SPSS Statistics for Windows, Version 25.0. Armonk, NY: IBM Corp).

## Results

A total of 194 invitations were sent to persons who were interested in participation, of whom 166 (86%) fully completed the questionnaire. After excluding 57 respondents based on the predefined exclusion criteria, a total of 109 (66%) questionnaires were used for analysis. Participant characteristics are displayed in Table 1. The mean age of the respondents was 52.8 years (SD 14.2). Fifty-seven persons (52%) had a known genetic defect, with SPG4 being the most frequently reported subtype (36/57 = 63%). Almost 50% of the participants had a disease duration of more than 15 years and more than 50% were unemployed, retired, or had a formally identified occupational incapacity. Almost 50% of the respondents reported micturation problems and used walking aids outdoors, whereas merely 12% was able to walk 100–1000 m outdoors without walking aids. The degree of motor disability and autonomic dysfunction (e.g. bladder dysfunction) have been reported in detail in our previous publication [5].

**Table 1:** Participant characteristics (N = 109)

Characteristics	n
Sex (men/women)	54/55
Age in years (Mean (SD))	52.8 (14.2)
Known genetic defect	57
SPG3a	4
SPG4	36
SPG5a	2
SPG7	3
SPG8	3
SPG10	2
SPG17	1
SPG31	5
SPG72	1
Affected first-degree relatives	83
Duration of spasticity symptoms (years)	
1–5 years	21
5–10 years	21
10–15 years	16
More than 15 years	51
Micturation problems	49
Use of electrical wheelchair outdoors	29
Use of walking aids outdoors	50
Walking ability between 100 and 1000 m without walking aids	13
Non-neurological comorbidities	
COPD	6
Diabetes	1
Hypertension	9
Joint disorders	13
Cardiac problems	4
Other	7
Employment	
Full-time work	27
Part-time work	14
Self-employed	8
Student	3
Retired	20
Unemployed	10
Occupational incapacity	27
Fully disabled (100%)	21
Mostly disabled (66–99%)	6

### Needs

**Fig. 1:**
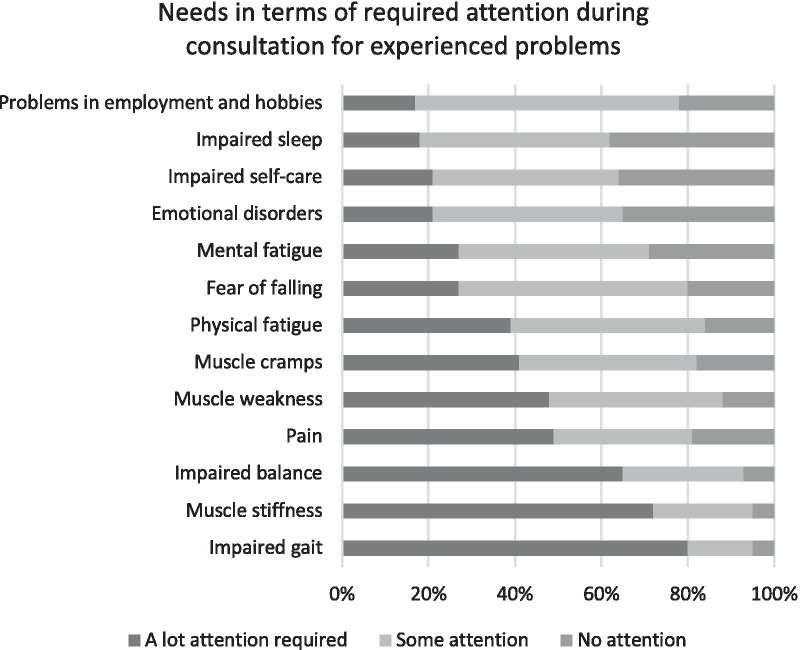
Participants' needs in terms of required attention during consultation for their experienced problems (N = 109)

Participants were asked to indicate the amount of attention (answering options: none, some, a lot) required during consultation with their physician for the healthcare needs regarding the experienced impairments and disabilities (see Fig. 1). The majority of the participants (80%) prioritized gait impairments, followed by muscle stiffness (72%) and impaired balance (65%), all requiring “a lot of attention” (answering options see Fig. 1). Other relevant topics were: pain, muscle weakness, muscle cramps, physical and mental fatigue, fear of falling, emotional problems, impaired self-care capacity, impaired sleep, and problems with employment and hobbies; all requiring minimally “some attention” in at least 60% of the participants.

### Expectations

The majority of the participants (63%) expected beneficial effects of the proposed interventions on gait (see Table 2). Other expectations, such as improved self-confidence, well-being, self-care, night rest, and reduction of pain and spasticity were less prevalent (< 33%), as were expectations related to enjoying leisure time and hobbies, returning to previous routines, employment, perform simple tasks, or drive a car. In the free entry fields, four participants stated that they did not have any expectations of the proposed interventions, whereas two participants had the expectation that treatment would slow down the progression of their disease.

**Table 2:** Expected effects of proposed interventions (N = 109)

Expected effect	% of participants
Improved gait	63
Improved self-confidence	32
Improved well-being	27
Enjoying leisure time and hobbies	26
Improved self-care	22
Returning to previous routines	20
Absence of pain	19
Improved night rest	15
Absence of spasticity	15
Being able to work	13
Better performance of simple tasks	12
Driving a car	9

### Healthcare professionals

Eighty-two percent of the participants had no or less than one consultation per year with a neurologist, whereas 16% visited a neurologist once or twice a year. The remaining 2% had three or more neurological consultations per year. As for consultations with a rehabilitation physician, 53% of the respondents had no or less than one consultation per year, 31% visited a rehabilitation physician once or twice a year, and the remaining 16% had three or more consultations per year.

Thirty-four percent of the participants had no consultations with a physiotherapist, 14% was treated by a physiotherapist once or twice per month, and 52% was treated more than three times per month. As for occupational therapy, 95% of the participants did not receive any consultations. Four percent visited an occupational therapist once or twice per month and 1% percent had more than three visits per month.

In everyday life, 49% of the participants did not need help from others, whereas others relied on the support from relatives (37%) or professional caregivers (12%). Two percent of the participants did not know how to organize support for the challenges they experienced in everyday life.

### Proposed interventions

Participants reported that a wide range of pharmacological and non-pharmacological interventions were proposed by their treating physicians (see Table 3).

**Table 3:** Interventions proposed by treating physicians (N = 109)

Proposed interventions by physician	% of participants
Non-pharmacological interventions	
Physiotherapy	81
Orthopedic footwear	55
Splints	28
Postural exercises	15
Massage	15
Psychological support	12
No intervention	10
Ankle–foot surgery	9
Hip surgery	4
Stress reduction	2
Education	2
Occupational therapy	2
Knee surgery	2
Social work	1
Osteopathy	1
Fatigue management	1
Lower spine surgery	1
Pharmacological interventions	
Oral spasmolytic drugs	41
Intramuscular botulinum toxin injections	26
Intrathecal baclofen	5

### Non-pharmacological interventions

Physiotherapy was by far the most often proposed non-pharmacological intervention (81%), followed by orthopedic footwear (55%) and splints (28%). Sometimes coaching and support (e.g. psychological support and stress reduction), education, occupational therapy, social work, and fatigue management programs were proposed. Orthopedic surgery was proposed to only a small number of participants.

### Pharmacological interventions

Oral spasmolytic drugs (41%) were most often proposed, followed by intramuscular botulinum toxin (BTX) injections (26%). To a small number of participants (5%), the possibility of intrathecal baclofen administration (ITB) was proposed. A combination of pharmacological interventions was advised to 14% of the participants, whereas 38% reported to have not been offered any spasmolytic treatment.

Pharmacological interventions were mainly focused on reducing muscle stiffness, muscle cramps, and improving balance and gait. If pharmacological interventions were proposed, 72% of the participants were informed by their physician about possible side-effects. Insufficient information about the pros and cons of oral medication was reported by 27%. For BTX injections and ITB these numbers were 8% and 20%, respectively.

Before receiving an intervention, 43% of the participants was subjected to an instrumented clinical gait analysis (including 3-D motion capture for kinematics, force plates for kinetics, and surface electromyography for muscle activation patterns). To those who were proposed orthopedic surgery, instrumented 3-D gait analysis was performed in 69% of the cases.

### Experienced pharmacological treatment effects

**Fig. 2:**
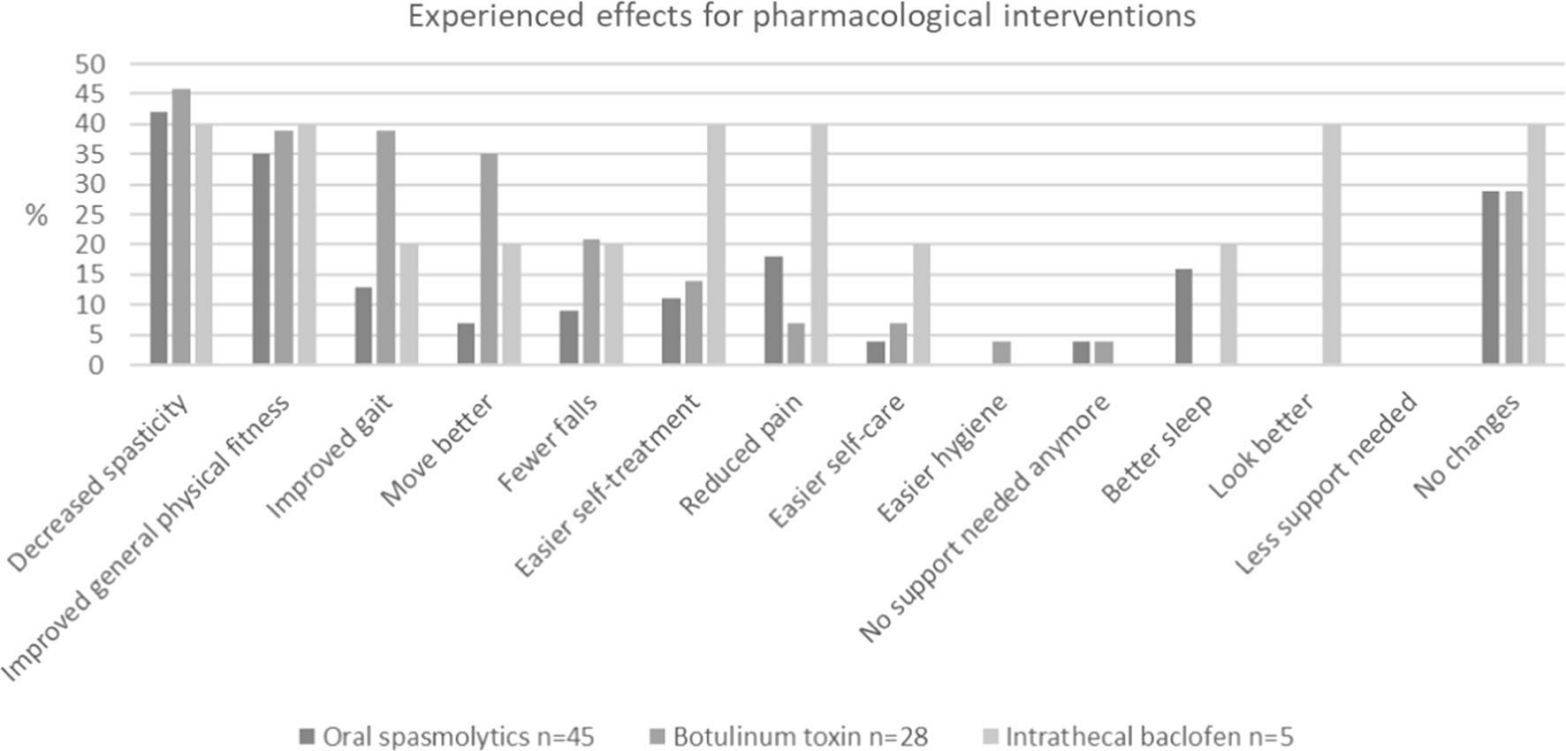
Experienced effects of oral spasmolytics (N = 45), botulinum toxin injections (N = 28), and intrathecal baclofen (N = 5)

Figure 2 provides an overview of the percentage of participants that experienced specific treatment effects of pharmacological interventions. Independent of the type of intervention, 35–46% of the participants experienced decreased spasticity and improved general physical fitness. Other experienced effects differed per type of pharmacological intervention. Improved gait and movement was more likely to occur after treatment with BTX injections (35–38%), compared to oral drugs (7–13%) or ITB (20–25%), whereas pain reduction and easier self-care and self-rehabilitation were most likely after ITB (20–40%). The experienced effects on falls reduction were best for BTX injections and ITB (20–21%). Sleeping responded best to oral drugs and ITB (16–20%). A substantial proportion of the participants experienced no effects at all from oral drugs (29%), BTX injections (29%), or ITB (40%).

Treatment with oral spasmolytic drugs was discontinued in 13% of the participants, mainly because of fatigue. Treatment with BTX injections was ceased in 7% of the participants, due to absence of effect. ITB was ceased in one of the 5 participants, because of too much muscle weakness.

**Fig. 3:**
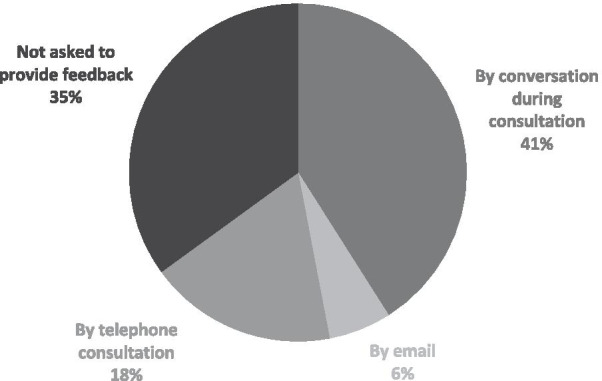
Methods of providing feedback on treatment effect

The effect of the pharmacological treatment was mainly evaluated during the subsequent consultation with the treating physician. Thirty-five percent of the participants reported that they were not asked for feedback regarding the effects of the intervention. Figure 3 shows the frequency of the methods used for providing feedback about the effects of the pharmacological interventions.

## Discussion

This survey investigated the healthcare needs, expectations, and utilization regarding spasticity management in a sample of 109 persons suffering from pure HSP in the Netherlands. We found that needs and expectations were primarily focused on relief of muscle stiffness and reduction of balance and gait impairments. Yet, many participants also expressed a need for relief of pain, muscle cramps, fatigue, and fear of falling as well as reduction of emotional problems, self-care and sleeping problems, and problems with employment and hobbies. Notably, participants’ expectations regarding the latter domains were less prevalent than their needs. As for the healthcare providers involved, rehabilitation physicians were consulted more often than neurologists, and physiotherapists much more than occupational therapists. Physiotherapy, orthopedic footwear, and splints were the most often proposed non-pharmacological interventions, whereas oral spasmolytic drugs were the most often administered pharmacological intervention, followed by intramuscular BTX injections. While both types of pharmacological intervention were experienced by participants to reduce spasticity and improve general physical fitness, improved motor control was deemed more likely after focal BTX treatment, whereas pain reduction and improved night rest were more likely to occur after systemic treatment (intrathecally more than orally).

### Healthcare needs and expectations

The participants’ needs as observed in this survey are in line with the results of our qualitative study amongst 14 persons with pure HSP [6]. In the current study, we were able to quantify these needs, showing that 65–80% of the participants asked for a primary focus on relief of lower-limb muscle stiffness and reduction of balance and gait impairments. Nevertheless, many participants (> 60%) also expressed the need for substantial clinical attention being directed at relief of pain, muscle cramps, fatigue, and fear of falling as well as a focus on improving societal participation and emotional well-being. Surprisingly, despite the fact that 49% of the respondents reported micturation problems (see Table 1), only 3% expressed the need of solving these problems. This apparent contradiction may be caused by the absence of a predefined answering option in the questionnaire. Therefore, in-depth exploration of individual patient needs—in conjunction with caregivers—may help to disclose needs in people with HSP that may otherwise remain unnoticed. In addition, because physicians and physiotherapists typically tend to pay more attention to sensorimotor symptoms than emotional and participation problems, our findings make a plea for a more interdisciplinary biopsychosocial approach to the clinical management of people with HSP, also involving professionals with a primary occupational and/or psychosocial orientation.

Remarkably, not many participants expressed expectations in the domains of non-motor symptom relief, self-care, returning to previous daily-life activities, or employment. This finding may be related to the fact that HSP is a progressive *inherited* disease [2] that forces patients to constantly adapt to slow increments in physical impairments and activity limitations. In this perspective, people with HSP are hoping to slow down physical deterioration rather than expecting improvement. This notion is supported by the finding that only 15% of our participants expected to ever be free of spasticity, while no more than 20% expected to be able to return to previous routines after treatment. These findings are in contrast to the results of an international survey amongst persons with various causes of *acquired* spasticity, such as stroke, multiple sclerosis and traumatic brain injury, of whom two thirds expected to achieve absence of spasticity [8]. This latter study also found a larger proportion of patients being treated with pharmacological interventions (73% BTX injections and 57% oral spasmolytic drugs) as well as more patients experiencing treatment effects (81% less muscle stiffness and 9% no effect). Apparently, there is a difference in expectations, proposed interventions, and experienced effects between persons with inherited progressive spasticity versus those with acquired chronic spasticity. Apart from the progressiveness of their spasticity, people with pure HSP have often witnessed relatives coping with progressive spastic paraparesis. Instead, people with acquired chronic spasticity, e.g. due to stroke, may have experienced regression of paresis and/or spasticity after disease onset. It is also possible that healthcare providers are more hopeful and willing to intervene in patients with acquired, non-progressive spasticity compared to those with inherited, progressive spasticity. Based on the current results and our clinical experience, we assume that the majority of people with pure HSP have rather realistic expectations with regard to effects of spasticity management, but are underinformed with regard to the possibilities of other forms of clinical management (e.g. occupational therapy, energy conservation management, psychosocial interventions, vocational advice). Our previous qualitative study has shown that—in this perspective—persons with HSP ask for adequate information and coaching by their healthcare providers [6]. Healthcare professionals should incorporate this knowledge in their treatment approach.

### Healthcare utilization and experienced treatment effects

Rehabilitation physicians tended to be consulted more frequently than neurologists by our participants. This observation is probably characteristic of the Dutch healthcare system. In the Netherlands, the diagnostic phase of HSP is typically led by neurologists, whereas spasticity management largely takes place within rehabilitation teams supervised by rehabilitation physicians. More interestingly, only a few participants had consulted an occupational therapist, despite the fact that limitations in activities and participation were frequently reported. This is remarkable given the fact that occupational therapy is easily accessible in rehabilitation centers, hospitals and community practices all over the Netherlands. In addition, costs of occupational therapy are reimbursed by all Dutch health insurances. Probably, there is unawareness amongst people with HSP as well as amongst their treating clinicians of the services and solutions that occupational therapists can provide. A similar explanation may underlie the low rate of consultation by professionals with psychosocial expertise. In neurological conditions such as Parkinson or stroke, occupational therapy has proven to be effective [9, 10]. In their qualitative study in 2013, Grose et al. concluded that persons healthcare professionals should be more aware of the emotional aspects of living with HSP [11].We believe that our data justify more involvement of both occupational therapists and psychosocially oriented disciplines in the clinical management of people with HSP.

Of all interventions, physiotherapy was most often provided, but our data do not allow any conclusions regarding the content or experienced effects of physiotherapy. More than half of the participants used orthopedic footwear, which may be related to a higher risk of ankle–foot deformities and ankle instability associated with HSP [12]. Splints were used by more than a quarter of all patients, probably to support lack of foot elevation and to prevent tripping. Despite limited evidence in the literature [13] and a frequent lack of effects experienced by almost one third of the users in the current study, oral spasmolytic drugs were used by 41% of our participants. In contrast, an almost equal number of participants (38%) had never been offered any spasmolytic treatment. Of those who did use oral medication, almost one third felt underinformed. These results seem to point towards a pharmacological area characterized by a variable level of quality of evidence, and sometimes contradicting guidelines and recommended monitoring tools [14]. The figures on informed consent with BTX and ITB treatment seem to be slightly better, but inferences regarding their experienced effect compared to oral spasmolytic drugs cannot be made due to selection bias and the (very) small numbers of people on these more invasive treatments. Considering the type of treatment effect, especially participants with an ITB pump seemed to have a greater likelihood of experiencing pain reduction, easier self-care and self-rehabilitation, and better sleep, whereas those who received BTX treatment seemed to have a higher chance of attaining improved motor control and capacity. But also in this respect, our results should be interpreted with caution.

Both the natural daily fluctuations in spasticity and the fluctuations induced by interventions such as botulinum toxin require careful communication between patients and healthcare providers to determine the optimal timing of (subsequent) interventions. Remarkably, more than one third of our respondents was not asked for any feedback on treatment results, whereas 41% was asked for feedback during subsequent consultations. To support patients in providing feedback, systematic monitoring of patient-relevant outcomes is crucial. It will help to tailor interventions to individual needs, to evaluate the effect of interventions, and to optimize timing [15].

### Strengths and limitations

A strength of this study is that we analyzed questionnaires from a relatively large group of people with pure HSP in the Netherlands, given the relatively low estimated prevalence of 800 persons with pure HSP in our country [16]. Our study sample also showed an equal sex distribution, a wide age range, and a large variation in duration of spasticity and underlying genetic defects, which underscores its representativeness for the Dutch population with pure HSP. As we included only participants with pure HSP, our findings cannot be generalized to people with more complicated forms of HSP. Another limitation is that our results cannot readily be generalized to people with HSP in other countries, given the differences in healthcare systems. However, since our results are in line with the findings of Grose et al., who studied the experiences of persons with HSP and healthcare professionals in England [11], we believe that a cautious use of our findings in the realm of Western European countries is warranted.

Another limitation is that only subjects without a positive family history or genetic diagnosis were checked for a formal diagnosis of pure HSP made by a neurologist. By including and excluding subjects based on specific questions, we tried to obtain a homogeneous sample with pure HSP, but it is possible that some people were incorrectly enrolled or excluded.

Our web-based survey was partly (category D) based on a previous international survey and extended with questions based on findings of a previous qualitative study [6] and input from representatives from the national patient organization. Yet, the involvement of healthcare professionals, who might have preconceptions regarding important aspects of HSP, might have biased its content. Furthermore, some questions allowed only dichotomous answers (yes /no), which might have influenced the relatively low scores for expectations regarding symptom relief (as participants could not indicate an expected partial relief). The fact that answers were given in complete anonymity and without any time limit are considered strengths.

### Conclusion

Based on this web-based survey in the Netherlands, there seems to be ample room for improvement to meet and attune the healthcare needs and expectations of people with pure HSP. Besides relief of their motor symptoms and incapacities, they express a clear need to address non-motor symptoms and functional limitations, for instance regarding pain, fatigue, emotional problems (i.e., lack of self-confidence, fear of falling), impaired sleep and self-care, and problems with occupation and participation. In addition, the provision of adequate information about pharmacological interventions seems to be insufficient for many patients to allow shared decision making. These conclusions warrant a more pro-active attitude of healthcare providers as well as a interdisciplinary approach for a substantial proportion of the HSP population, also involving professionals with a primary occupational and/or psychosocial orientation. Regarding content of spasticity management in this population, there is a need for (inter)national guidelines, given the variety of clinical practice and the sparsity of clinical evidence. Hence, we recommend that future research aims at both improving spasticity management and broadening the scope of clinical management in people with pure HSP.

## Supplementary Information

**Additional file 1**. Variables and answer options survey.

## Data Availability

All data generated or analysed during this study are included in this published article [and its supplementary information files].
